# Human Immunodeficiency Virus-Associated Tuberculosis

**DOI:** 10.1155/2011/513967

**Published:** 2011-12-19

**Authors:** Katalin A. Wilkinson, Stephan Schwander, M. Estee Torok, Graeme Meintjes

**Affiliations:** ^1^Clinical Infectious Diseases Research Initiative, Institute of Infectious Diseases and Molecular Medicine, University of Cape Town, Observatory 7925, South Africa; ^2^Division of Mycobacterial Research, MRC National Institute for Medical Research, London NW7 1AA, UK; ^3^Department of Environmental and Occupational Health and Center for Global Public Health, School of Public Health, University of Medicine and Dentistry New Jersey, 683 Hoes Lane West, Piscataway, NJ 08854, USA; ^4^Department of Medicine, University of Cambridge, Addenbrooke's Hospital, Cambridge CB2 0SP, UK

The human immunodeficiency virus (HIV), causing the acquired immunodeficiency syndrome (AIDS), was first reported thirty years ago. The UNAIDS published the latest statistics on the global HIV and AIDS epidemic in November 2010 and estimated that in 2009 there were 33.3 million people living with HIV/AIDS worldwide (Worldwide HIV and AIDS statistics, http://www.avert.org/worldstats.htm). While the annual number of new HIV infections has steadily declined, and the number of people receiving antiretroviral therapy increased, the HIV pandemic has so far caused the death of nearly 30 million people from AIDS-related causes. The sub-Saharan African region carries the greatest burden of this pandemic, with 22.5 million adults and children living with HIV/AIDS in 2009. In South Africa alone, there are an estimated 5.6 million HIV-infected people, more than in any other country, with almost one in three women aged 25–29, and over a quarter of men aged 30–34, living with HIV (HIV and AIDS in South Africa, http://www.avert.org/aidssouthafrica.htm).

Tuberculosis (TB) is an important public health problem representing the most frequent opportunistic infection in HIV infected persons globally. In 1993, the World Health Organization (WHO) declared TB a global public health emergency, when an estimated 7-8 million cases and 1.3–1.6 million deaths occurred each year. Today, TB remains a leading cause of death in low- and middle-income countries, and the latest WHO report on global TB control indicates that there were an estimated 8.8 million incident cases and 1.4 million deaths from TB in 2010 (http://www.who.int/tb/en/). HIV infection is the greatest risk factor for acquiring *Mycobacterium tuberculosis (M.tb)* infection and developing TB. The devastating association between HIV and TB means that up to 1.2 million (12–14%) of the new TB cases were amongst HIV-infected people in 2010, and TB caused the death of an estimated 0.35 million HIV-infected people. The proportion of HIV-*M.tb*-coinfected persons is highest in Africa, with the African Region accounting for overall 82% of TB cases among people living with HIV. 

The risk of TB is increased during all stages of HIV infection from about 10% over a lifetime (in HIV-uninfected individuals) to as high as 30% per annum in patients with advanced HIV infection [1, 2]. These circumstances define the analysis of the immune response to TB in the context of HIV infection as a pressing research priority. This special issue is therefore devoted to HIV-associated TB. It comprises three review papers and six research papers. 

The first paper is a general overview of HIV-associated TB and focuses on the intersecting HIV and TB epidemics in countries with a high burden of both infections. Among the many challenges, the authors discuss the diagnosis of TB in HIV-infected patients, difficulties posed by antiretroviral drug interactions when treating HIV-*M.tb*-coinfected patients, and the WHO-recommended interventions collectively known as collaborative TB/HIV activities, such as HIV testing in TB patients, integration of HIV and TB services, provision of isoniazid preventive therapy (IPT), infection control, minimizing airborne transmission, and managing recurrent TB. 

The crucial question of when to initiate combined antiretroviral treatment (cART) in relation to TB treatment, balancing the increased risk of immune reconstitution inflammatory syndrome (IRIS) associated with early cART with the mortality associated with delaying HIV treatment, is discussed in the second review paper. TB-associated IRIS (TB-IRIS) is an important early complication of cART, reflecting the fact that the immune responses to TB may contribute to both protection and pathology. Two forms of TB-IRIS are recognized: (1) paradoxical, that occurs in patients who are established on TB treatment before cART, and manifest with recurrent or new symptoms and clinical features of TB after initiation of cART; (2) unmasking TB-IRIS, that has been defined in patients who are not receiving treatment for TB when cART is started, but present with active TB within 3 months of starting cART and show a heightened intensity of clinical manifestations with a marked inflammatory component [3, 4]. The authors conclude that there is compelling evidence that initiation of cART should not be delayed in HIV/TB-coinfected individuals. The WHO recommends the initiation of cART between 2 and 8 weeks subsequent to the initiation of TB therapy in individuals with CD4 counts <200/*μ*L. Three publications (published since this review paper) of clinical trials in patients with HIV-associated TB [5–7] describe survival benefits and increased AIDS-free survival resulting from early cART initiation after the start of TB therapy, particularly in persons with far-advanced immunosuppression (CD4 <50/*μ*L), while indicating that the risk of IRIS was higher with earlier than with later cART initiation. This survival benefit of early cART occurs despite the higher risk of IRIS in those started earlier and in those with a CD4 count <50/*μ*L, and despite the fact that IRIS itself can be associated with mortality (case fatality rate of 3.2% in a recent meta-analysis) [8]. Although guidelines have been developed for resource-limited settings (3), the need to detect and appropriately treat IRIS in a timely fashion outside of a controlled clinical study environment remains a major challenge and requires further exploration.

The third paper discusses the concept of “latent tuberculosis infection” in HIV-infected individuals. Immunological tests, such as tuberculin skin test (TST) and interferon-gamma release assays (IGRAs) provide evidence of *M.tb* sensitization in one-third of the global population. Thus, more than two billion individuals are estimated to be latently infected with *M.tb* and show no clinical symptoms but live with the risk of subsequent progression to overt clinical disease, particularly in the context of coinfection with HIV. The traditional paradigm that distinguishes latent *M.tb* infection from active TB as two distinct compartmentalized states appears to be too simplistic. Recently, it has been suggested that clinically defined latent *M.tb* infection actually represents one extreme on a spectrum (of immune responses, mycobacterial metabolic activity, and bacillary numbers) that runs from elimination of live bacilli to subclinical and clinical symptomatic disease [9, 10]. The authors of this paper propose that the impact of HIV infection on this spectrum might be better conceptualized as a shift from controlled infection towards poor immune control, higher mycobacterial metabolic activity, and greater organism load, with subsequently increased risk of progression to active disease and discuss the evidence for such a model as well as the implications for interventions to control the HIV-associated TB epidemic.

The six research papers and clinical studies in this special issue follow the reviews in thematic order. Thus, the fourth paper describes HIV-associated TB mortality and predictors of death and survival in a tertiary care center in Brazil; the fifth and sixth describe the paradoxical consequences of dual HIV and TB treatment (TB-IRIS) in Northern India and Uganda and their predictors, followed by approaches to diagnosing *M.tb* infection and TB in HIV-infected people using existing immunodiagnostic (IGRA) and proposed methods (antigen 85 reactivity). The final paper focuses on genetic factors (HLA-B*∗*57) and gender influencing the incidence of TB in HIV-infected persons in Bangalore, India. 

This issue shows that progress is being made in unravelling the spectrum of protective and pathological immune mechanisms in HIV and *M.tb*-coinfected persons, and the approaches and consequences of their treatment. Indeed, WHO in its 2011 report presents encouraging trends of globally declining TB incidence rates at a time of broadening therapeutic options, TB vaccine candidates in phases II and III trials, and the development of fast and precise PCR-based diagnostic tools. However, major obstacles such as poverty and its consequences and availability of and access to health care impact the HIV and *M.tb* pandemics, while at the same time new attention is also needed on diabetes, cigarette smoking, and air pollution as risk factors in TB endemic areas. Bold goals such as “Zero Deaths” [11], increased disease-specific funding for treatment programs, development of new diagnostics, vaccines, and biomedical and clinical progress as presented here will be needed to sustain accomplishments and further decrease the HIV and TB impact on global public health. 

**Katalin A. Wilkinson** **Stephan Schwander** **M. Estee Torok** **Graeme Meintjes**

## References

[1] Badri M, Wilson D, Wood R (2002). Effect of highly active antiretroviral therapy on incidence of tuberculosis in South Africa: a cohort study. *The Lancet*.

[2] Lawn SD, Badri M, Wood R (2005). Tuberculosis among HIV-infected patients receiving HAART: long term incidence and risk factors in a South African cohort. *AIDS*.

[3] Meintjes G, Lawn SD, Scano F (2008). Tuberculosis-associated immune reconstitution inflammatory syndrome: case definitions for use in resource-limited settings. *The Lancet Infectious Diseases*.

[4] Meintjes G, Rabie H, Wilkinson RJ, Cotton MF (2009). Tuberculosis-associated immune reconstitution inflammatory syndrome and unmasking of tuberculosis by antiretroviral therapy. *Clinics in Chest Medicine*.

[5] Abdool Karim SS, Naidoo K, Grobler A (2011). Integration of antiretroviral therapy with tuberculosis treatment. *The New England Journal of Medicine*.

[6] Blanc FX, Sok T, Laureillard D (2011). Earlier versus later start of antiretroviral therapy in HIV-infected adults with tuberculosis. *The New England Journal of Medicine*.

[7] Havlir DV, Kendall MA, Ive P (2011). Timing of antiretroviral therapy for HIV-1 infection and tuberculosis. *New England Journal of Medicine*.

[8] Muller M, Wandel S, Colebunders R, Attia S, Furrer H, Egger M (2010). Immune reconstitution inflammatory syndrome in patients starting antiretroviral therapy for HIV infection: a systematic review and meta-analysis. *The Lancet Infectious Diseases*.

[9] Young DB, Gideon HP, Wilkinson RJ (2009). Eliminating latent tuberculosis. *Trends in Microbiology*.

[10] Barry CE, Boshoff HI, Dartois V (2009). The spectrum of latent tuberculosis: rethinking the biology and intervention strategies. *Nature Reviews Microbiology*.

[11] Keshavjee S, Harrington M, Gonsalves G, Chesire L, Farmer PE (2011). Time for zero deaths from tuberculosis. *The Lancet*.

